# An Inclusive Framework for Collaboration between Midwives and Traditional Birth Attendants and Optimising Maternal and Child Healthcare in Restricted Rural Communities in South Africa: Policy Considerations

**DOI:** 10.3390/healthcare12030363

**Published:** 2024-01-31

**Authors:** Maurine Rofhiwa Musie, Fhumulani Mavis Mulaudzi, Rafiat Anokwuru, Nombulelo Veronica Sepeng

**Affiliations:** 1 Department of Nursing Sciences, Faculty of Health Sciences, University of Pretoria, Pretoria 0084, South Africanombulelo.sepeng@up.ac.za (N.V.S.); 2 Department of Maternal and Child Health, llishan School of Nursing, Babcock University Remo, Ilishan-Remo 121003, Nigeria; anokwurur@babcock.edu.ng

**Keywords:** collaboration, framework, midwives, nominal group technique, stakeholder engagement, traditional birth attendant

## Abstract

Collaboration between midwives and traditional birth attendants for maternal and child healthcare is a challenge in rural South African communities due to the absence of a guiding framework. To address this, this study sought to develop and validate an inclusive framework informed by the Donabedian structure–process–outcome (SPO) framework for collaboration between these healthcare professionals. Method: Key stakeholders were invited to participate in a co-creation workshop to develop the framework. Twenty (20) participants were purposively sampled based on their maternal and child healthcare expertise. A consensus design using the nominal group technique was followed. Results: Participants identified the components needed in the framework, encompassing (i) objectives, (ii) structures, (iii) processes, and (iv) outcomes. Conclusion: This paper will contribute to the development of an inclusive healthcare framework, providing insights for stakeholders, policymakers, and practitioners seeking to improve maternal and child healthcare outcomes in resource-constrained, rural settings. Ultimately, the proposed framework will create a sustainable and culturally sensitive model that optimises the strengths of midwives and TBAs and fosters improved healthcare delivery to rural South African communities.

## 1. Introduction

Globally, particularly in South Africa (SA), there is an increased interest in pregnant women accessing maternal and child healthcare services from both the Western and traditional health systems [1,2]. Thus, fostering collaboration between these health systems so they can complement each other is desirable [1]. Currently, there is a minimal collaboration among practitioners and a lack of explicit guidance on the integration of traditional birth attendants (TBAs), also known as traditional midwives or indigenous midwives, into the formal healthcare system [2,3]. Moreover, the lack of a framework to facilitate collaboration between midwives and TBAs for maternal and child healthcare poses a challenge in rural communities in SA. The World Health Organization (WHO)’s [4] recommendations on optimising lay health workers and the WHO’s [5] framework for working with individuals, families, and communities have recognised the need to specify the responsibilities of TBAs for maternal and newborn health (MNH) in low- and middle-income countries (LMICs), wherein they are an essential source of childbirth care. Childbirth is seen not only as a physical occurrence but also as a social event where tradition, culture, spiritual beliefs, and rituals converge, and these aspects are exemplified in the practices performed by TBAs or midwives [6]. The WHO Alma Ata Declaration (1978) adds that TBAs are found in most local communities; therefore, it is worth exploring the possibilities of training and engaging them in primary healthcare [6,7]. 

In contrast, midwives are trained as skilled birth attendants (SBAs) with expertise in assisting pregnant women throughout labour and childbirth, and they are in the best position to train and work with TBAs, who are closer to the women in these communities [2,8,9]. In rural communities, TBAs provide access to maternal and neonatal healthcare services in ways that are culturally appropriate and respectful of religious beliefs [8]. Furthermore, they link women, families, and communities to the formal healthcare system [1,10].

The WHO has urged its member states to prepare legislation to govern the practice of traditional medicine [11,12]. The South African government’s promulgation of the Traditional Health Practitioners Act, No. 35 of 2004 (amended as the THP Act 22 of 2007) is the culmination of such efforts [1,13]. SA faces pluralism in healthcare, wherein the Western/formal healthcare system is regarded as dominant and co-exists with the traditional/indigenous system [13,14]. SA’s healthcare system is largely inefficient and unequal. Despite various areas of growth since 1994, gaps in wealth and health among the population are significant [15]. Some members of the population have the means to access first-world healthcare through the private sector. At the same time, many people who cannot afford this service are left to rely on governmental hospitals and clinics.

In comparison, government institutions are largely unreliable and fail to offer adequate specialist services. As a result, the country is planning reforms that aim to achieve universal health coverage (UHC) through the introduction of national health insurance (NHI) and primary healthcare (PHC). In 2011, the Minister of Health proposed a universal healthcare system accessible to all residents via a single fund that would cover everyone. However, currently, pregnant women receive free maternal healthcare services in primary healthcare settings [15,16].

Collaboration entails both health systems complementing each other. It involves working together for a common goal, such as ensuring high-quality healthcare for childbearing women [1]. Moreover, task shifting and knowledge-sharing emanate from collaboration, and with partnership, better referrals can be achieved, leading to improved health in the communities [2,17,18]. Collaborations can manifest in various ways within healthcare systems, which can be classified into four distinct types: integrative, inclusive, monopolistic (exclusive), and tolerant. A monopolistic system recognises only the Western health system and severely restricts all other practices [2,13,14,17]. Tolerant health systems are characterised by a predominant reliance on Western medicine within the national healthcare framework while simultaneously accommodating and regulating specific traditional practices [2,13,14]. On the other hand, an inclusive system recognises traditional practices but has not yet integrated them into all aspects of healthcare (e.g., delivery, training, education, and regulation). Lastly, an integrative system incorporates both traditional and Western health practices; this system is characterised by synthesising all healthcare practices available to optimise healthcare for all [2,13,14,17].

In an effort to promote facility delivery through skilled attendance and in light of the United Nations (UN) sustainable development goals (SDGs), the delivery of maternal and child healthcare by TBAs has been prohibited [8,18]. Some countries, like Nigeria, Ghana, and Indonesia, have successfully integrated TBAs into the local health system [18,19]. Strategies to promote skilled birth attendants typically entail establishing new roles for TBAs, boosting collaborations or teamwork, or connecting TBAs to formal health services [1,18]. Throughout the 1970s and 1980s, the WHO encouraged TBA training to improve maternal and child health to the extent of recommending the expansion of their role in other aspects of primary healthcare, such as vaccination programmes and malaria prophylaxis in Nigeria [6]. The main aim of introducing TBA training was to reduce the high level of maternal mortality. Despite training the TBAs, statistics still indicated high maternal mortality rates associated with TBA care. The literature argues the unsuccessful training of TBAs may be related to culture, didactic, and biomedical failure to consider traditional midwives’ knowledge and learning styles [6]. In Uganda, the “Partnership paradigm (PP) was implemented to promote mutual respect and accommodation between the formerly opposed biomedical staff and the TBAs” [6]. The main aim of the PP model was to build ongoing dialogue between the health professionals who would learn each other’s practices and techniques for better maternal and child healthcare. In Uganda and Indonesia, TBAs play a variety of roles, such as supporting and encouraging women to seek prenatal and postnatal care, as well as caring during childbirth. They also play broader roles in community-level health education and community mobilisation strategies to improve maternal and newborn health [20,21]. Developing cooperative relations through the mutual exchange of traditional and professional knowledge and emphasising such collaboration in in-service midwifery training are some strategies to increase collaborations. One of the challenges in developing a cooperative relationship between the midwives and the TBA is the lack of a conceptual framework determining the extent of the collaboration. The framework will serve as a guide for both midwives and TBAs [1,20,21]. Despite this, the literature review is silent regarding the conceptual framework for inclusive collaboration between midwives and traditional birth attendants, optimising maternal and child healthcare in rural-restricted communities in South Africa, using Donabedian model features.

### 1.1. SPO Donabedian Framework

The Donabedian SPO framework was adopted as a prototype, as its features are structure, process, and outcome [22,23,24,25].

#### 1.1.1. Structure

According to the Donabedian SPO framework, the structure entails the setting in which it is necessary to have resources and personnel to provide the recommended care [25,26]. Personnel refers to agents or items in the structure responsible for carrying out the required activity. The structure is defined as a setting in which healthcare is provided (e.g., facilities, equipment, numbers, and the qualifications of personnel) [25,26]. Based on the conceptualisations of the findings of the study, the following stakeholders were involved in the nominal group technique, such as midwives, traditional birth attendants, the Director of the National Directorate of Traditional Medicine, policymakers, labour organisations, and representatives from sub-Saharan countries.

#### 1.1.2. Processes

According to Donabedian [25], the processes follow the structure. Processes refer to what is done in providing and receiving care. This includes the rules, techniques, protocols, and activities required to achieve the outcome. As described, the findings of the study allude to the processes, which refers to the required activities to achieve collaboration between the midwives and traditional birth attendants for maternal and child healthcare.

#### 1.1.3. Outcome

According to Donabedian, outcome refers to the consequence of the provided healthcare (e.g., health status, satisfaction, and costs) [24,25,26,27]. It is also described as the activity’s final output. In the study, the findings envisage that the final results of the collaboration would foster culturally appropriate maternity care, guided by the synergy between the traditional and Western health systems. The Donabedian framework eloquently states, “A good structure increases the likelihood of good process, and good process increases the likelihood of good outcomes” [28]. The framework was deemed suitable, as it covers all relevant aspects of an organisation’s structure, process, outcome, and interrelations. For collaboration to succeed, the interaction between the categories should be bidirectional, and it is not a simple separation between cause and effect [29]. Studies have used Donabedian to develop a conceptual framework that can be used to optimise healthcare services in different populations [27]. However, this study’s findings will inform policy formation. Thus, the aim of this paper is to develop a conceptual framework for inclusive collaboration between midwives and traditional birth attendants, optimising maternal and child healthcare in rural South African communities using the Donabedian model.

## 2. Materials and Methods

### 2.1. Study Design

The nominal group technique (NGT) was employed to enable engagement with key stakeholders across Africa. We defined the key stakeholders as those with knowledge and years of experience in maternal healthcare services, researchers in the field, and the collaboration between traditional birth attendants and midwives. The consensus method (NGT) identifies strategic problems and develops appropriate and innovative interventions to address them [28,29]. Furthermore, NGT is a structured group-based technique to build consensus among the identified stakeholders [30]. The NGT process is commonly applied to homogenous groups and involves five phases: (i) Introduction and explanation; (ii) nominal or silent generation, where the participants consider their responses to questions and write them down. (iii) round-robin, where individual participants take turns to share their responses with the group; (iv) discussion and clarification, where the group elaborates on their responses—during this phase, items with similar meanings are grouped; and (v) voting phase, where each participant is asked to prioritise the listed items by assigning ranks to them. The ranking results are then collated into one list of priorities guided by the SPO model, which was used to develop the framework [28,30]. The process is discussed in detail below in Table 1:

### 2.2. Study Participants

We invited key knowledgeable stakeholders on midwifery and African traditional care from various settings to participate in the nominal group consensus workshop. The NGT team comprised a total sample of twenty (20) participants, including two midwives, three midwifery educators from nursing education institutions teaching maternal and child healthcare in SA, as well as one midwifery educator from Swaziland, and one researcher from Ghana with collaboration between the midwives and traditional birth attendants existing in their country. It also included one researcher from Nigeria, one policymaker from the National Directorate of Traditional Medicine, two policymaker directors from the National Department of Health (Maternal and child health), one from the community representatives of civil society, one from UNICEF, two TBAs, two labour organisation representatives (midwives and traditional health organisations), one student, one ethnographic healing professional from complementary alternative medicine, and one representative from the Society of Midwives in SA. The detailed characteristics are presented in Table 2.

### 2.3. Sampling Strategy

A non-probability purposive sampling strategy was used to select the participants in the study [29]. This sampling technique involves identifying and selecting participants with practical knowledge and experience in maternal healthcare.

### 2.4. Data Collection Procedure

The PI (M.R.M) facilitated the workshop with the help of a trained research assistant who acted as moderator for the session. The NGT process was broadly conducted in five phases during the four-hour workshop in November 2023. Due to the COVID-19 restrictions on travelling, the workshop was hybrid. The participants from Nigeria, Swaziland, and Ghana joined in on the online Teams platform. The other participants from SA attended the workshop face-to-face at the University of Pretoria in the Future Africa conference centre. Prior to the commencement of the workshop, the PI handed out the day’s programme and gave a brief report on the findings from Phases 1 and 2 of the study, conducted with the midwives and TBAs from various resource-restricted communities. The main aim of the NGT was to bring together key stakeholders’ ideas on how a collaboration framework may be developed between TBAs and midwives. The PI also shared the purpose of the meeting, which is for the key stakeholders to co-create a framework of collaboration for midwives and TBAs in SA to inform policy formation according to the SPO Donabedian framework.

Prior to collecting data, the researcher obtained written consent from the stakeholders invited to the workshop [30,31]. At the opening session, the stakeholders were given an opportunity to introduce themselves, their current positions, and their years of experience within the maternal and child healthcare field. Each participant received a welcome bag, including the day’s programme, flip charts, A4 paper, pens, coloured stickers, and a consent form document. The PI (M.R.M) posed the following central question to the group: What should a framework for collaboration between the midwives and TBAs for maternal health care services in South Africa entail? The researcher explained that the nominal group technique steps would be followed to elicit their ideas on how a framework for collaboration may be developed guided by the SPO Donabedian framework [28]. The 5 steps of the NGT that were followed to guide the workshop included step 1: introduction and explanation of processes to be followed; step 2: generation of ideas; step 3: round-robin; step 4: discussion; and step 5: voting. Before starting the workshop, the researcher asked the participants if they had any questions relating to the findings of Phase 1 that were presented. The more in-depth information on the processes followed is explained in Table 1:

### 2.5. Data Analysis

Data analysis in NGT is an ongoing process that involves ranking ideas and a thematic analysis of qualitative data [31]. This method was employed in this study, where the quantitative data obtained from the participants ranking ideas on a scale of 1–5 was analysed through the summing of votes allocated to each idea. The overall priority score for each theme was then calculated. This was done by capturing ranking responses into Google Forms and calculating the overall priority scores. A priority list of responses was then drawn and presented to the broader group. For the qualitative data analysis, the collected notes from the participants were used under the identified themes according to the SPO Donabedian model.

### 2.6. Ethical Considerations

The Research Ethics Committee in the Faculty of Health Sciences at the University of Pretoria granted consent for the study (ethics number 597/2020). The Gauteng Province National Department of Health was also approached for approval and was granted; further approval was also obtained from the City of Tshwane Municipality. The stakeholders voluntarily agreed to participate in the study and signed a written consent form to participate.

## 3. Results

### 3.1. Characteristics of Study Participants

The NGT team comprised 20 participants from ages 27–60. All the stakeholders in the workshop reported involvement in maternal and child healthcare, and their specific roles are reported in Table 2.

### 3.2. Nominal Group Ranking

**Figure 1:**
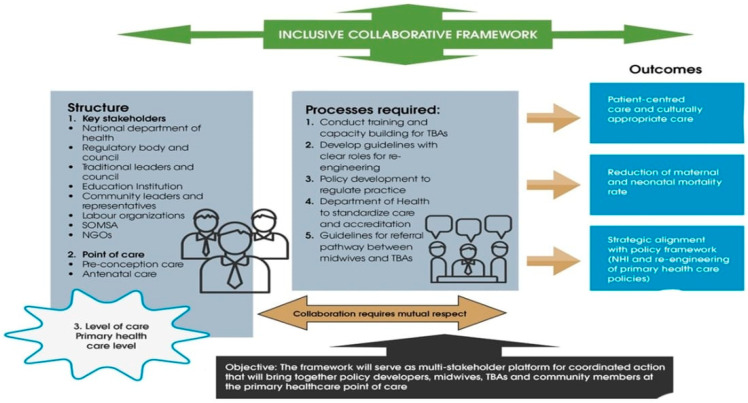
Inclusive framework for collaboration between midwives and traditional birth attendants for maternal and child healthcare.

The stakeholders identified certain aspects that were later ranked for inclusion in the framework guided by the SPO Donabedian framework. The stakeholders had to first agree on the type of collaboration that may exist between the midwives and TBAs. The results highlight that (56%) fifty-six per cent of the stakeholders in the NGT workshop agreed that the framework should follow the inclusive healthcare system. The inclusive healthcare system is defined as “recognising traditional practice, but not yet fully integrating their practice within the aspects of health care” [14]. Furthermore, the collaboration can be in the form of training and education of traditional birth attendants and to regulate their practice by developing policy directives [18]. The following results emanated according to the SPO framework, which can be referred to in Figure 1.

#### 3.2.1. Structure

The structure–process–outcome (SPO) Donabedian framework (1988) refers to the structure as the demographic of the required key stakeholders or role-players to collaborate. Within the consensus engagement (76%), participants agreed that the following structures would be implemented to support the collaboration between the midwives and TBAs: (i) The different key stakeholders, (ii) the role they will play in the collaboration, (iii) point of care (types of services provided, such as pre-conception and antenatal care), and (iv) the level of care (primary level, community level, regional level, and tertiary level) in which the collaboration will exist.

Most (75%) stakeholders agreed that the objective of the inclusive collaborative framework should:

*“The framework will serve as a multi-stakeholder platform for coordinated action that will bring together policy developers, midwives, TBAs and community members at the primary healthcare point of care”.*

All stakeholders

(i, ii) Key stakeholders or role-players

It was agreed that the following key stakeholders are required for the collaboration and to be fully engaged in the collaboration, as depicted in Table 2, and are supported by the following quote.

*“The National Department of Health as policymakers will be responsible for policy development and ensuring relevant guidelines are developed to facilitate the collaboration between traditional birth attendants and midwives”.*

In addition, another role includes:

*“The education institutions will be responsible for facilitating the training and developing the curriculum for the traditional birth attendants”.*

(iii) Point of care

Point of care is described as the different stages of maternity care in which the TBAs can play a role. The majority (88%) of stakeholders agreed that the collaboration of midwives and traditional birth attendants should be for only pre-conception and antenatal points of care.

*“During antenatal care, the traditional birth attendants can be responsible for screening for pregnancy-related problems, assessing and referring high risk women to the antenatal clinics and providing information to the women to prepare for childbirth”.*

(iv) Level of care

For the level of care, the stakeholders reached a consensus that the collaboration should exist at the primary healthcare level. DoH [30] defines the primary healthcare level as the clinic level where the health facility normally functions on weekdays, and the midwives working in PHC are responsible for low-risk and intermediate-risk women for antenatal care; in case of complications, the women are referred to the appropriate health facility.

However, 78% of stakeholders agreed that TBAs should be integrated into the healthcare system at the primary healthcare level.

*…. Within the primary healthcare level, the TBAs can manage low-risk antenatal care women and refer them to the nearest clinic when dangers and problems are encountered during pregnancy”.*

Another participant added that:

*“We work almost the same as the community workers that visit the women at home; we also either make home visits or ask the mother to come to the indaba (sacred healing hut) after homebirths and being seen in the hospital. But mostly, we need to volunteer as traditional birth attendants to work as part of the ward-based teams”.*

TBA

#### 3.2.2. Process

The results of the paper highlight the processes according to the SPO Donabedian framework. The process is defined as the actions that need to be taken during the collaboration [22]. The stakeholders identified 13 processes that can be followed during the collaboration. From the 13 processes, the top five priorities were ranked. The participants reached a consensus on the following five highest-rated processes to the lowest that need to be followed in this framework, as indicated in Table 3 below and the following extracts.

*“The regulatory councils of the traditional birth attendants (Traditional Health Organisation) and the midwives (South African nursing council) are responsible for regulating and registering the practitioners once the policy to govern their practice has been implemented”.*

#### 3.2.3. Outcomes

According to the SPO framework, outcomes refer to the consequences or positive attributes of the actions done [22]. The consensus was reached on the three highest priority outcomes that should be achieved by the framework during the collaboration, as indicated in Table 4 below.

The following quote supports the culturally appropriate care rendered by TBAs:

*“Pregnant women respect the care we provide to them during pregnancy as it is according to their culture and traditional beliefs”*

TBA

In support of the reduction in maternal and neonatal mortality rates:

*“If we work together, we can save more lives and help a lot of mothers that are dying during birth”*

TBA

## 4. Discussion

This paper has identified the key areas for developing the inclusive, collaborative framework for midwives and TBAs on maternal and child healthcare services in rural South African communities. From the highest-ranked priorities, the paper calls for broader policy implications of the proposed inclusive collaborative framework. It advocates for policy changes that officially recognise and integrate TBAs into the formal healthcare system [1,15,16]. The article suggests that policymakers should consider developing guidelines that promote collaboration, ensuring that regulatory frameworks support and facilitate this inclusive approach to maternal healthcare [32]. Likewise, in Ghana, the TBAs are integrated into the community health worker interim to train and equip them to perform uncomplicated deliveries, identify obstetric complications, and refer them to community healthcare nurses for further management [6,33]. In Nigeria, unilateral collaboration exists, creating a dichotomy between the traditional and biomedical health systems around pregnancy and birth. The country also reported that most births still occur under the care of TBAs. The reasons given by women for their preference for TBAs’ care include accessibility, affordability, and provision of compassionate, culturally competent, and acceptable care [34]. In support, the TBAs in Kenya also indicated that women prefer their services for the above reasons. Individuals frequently use both systems simultaneously, depending on their health needs. However, because most modern healthcare facilities are located in cities, the traditional healthcare system is frequently the most accessible and economical for people in rural areas of the country [34].

Some studies indicate that the TBAs are still not formally integrated into the health system [33,35]. In Nigeria, there is no regulatory body for TBAs, and statistics are lacking in relation to the exact number of TBAs practising in the country. However, what is known is that they offer reproductive services, including infertility, antenatal, intrapartum, and postnatal care, as well as treatment of threatened miscarriage [1,33]. Some communities in SA do not explicitly use the concept of a traditional birth attendant; instead, they refer to them as women who assist other women during birth or traditional health practitioners [11]. TBAs are usually older women who learn skills from their seniors and are appreciated in society for their knowledge and experience [32]. However, in SA, the practice of traditional birth attendants is not officially supported and is not integrated into the formal healthcare system. Current policies present a separation between professional midwives and traditional or community midwives, leading to midwives’ integration into a hierarchical, intensely colonialist system that has doctors at the top, professional midwives in the middle, and community midwives at the bottom, with no power and very little government support [36]. In this system, doctors have most of the power. Professional midwives, who are usually biomedically trained, often buy into this hierarchy and work to impose biomedical models of birth on indigenous populations [36]. Unfortunately, in some settings, the traditional midwives were receiving blame for the high rates of maternal mortality when, in fact, most of the deaths occurred among women who had given birth in the hospital as a result of complications from caesarean sections [34,36].

Secondly, in terms of the structures that should be involved to facilitate the collaboration between midwives and TBAs, the findings advocate for including the TBAs in only offering antenatal services. Collaborative frameworks must recognise and respect traditional practices, beliefs, and community norms. Studies from Ghana and Nigeria suggest that most African women consult traditional health practitioners during pregnancy to strengthen their pregnancy and to receive traditional medicine that will protect them from any dangers and thus refrain from attending antenatal care during this time due to the associated beliefs surrounding pregnancy [1,33]. Thus, when collaboration exists between traditional birth attendants and midwives, they will be able to identify the women at risk and refer them to clinics on time. Some of the literature indicates that pregnant women do not go to clinics because of the hospital staff’s attitude they experience when visiting these clinics. Women fear being scolded and shouted at for late clinic bookings [33,34]. TBAs should be included at the primary health level, where they are included in the ward-based outreach teams (WBOT) according to the three teams indicated for re-engineering. According to Alma Ata, the primary healthcare package aims to “address the main health problems in the community through provision of promotive, preventative, curative and rehabilitative services” [7,35]. The first point of contact in rural communities is the traditional birth attendants; thus, this study proposes the inclusion of TBAs in the WBOT teams [1]. The ward-based outreach teams in SA comprise a professional nurse, one health promoter, one environmental health officer, and six leading community healthcare workers (CHWs). This team is responsible for offering community-based services in the community. TBAs are an available cadre that can add to the service provision and attainment of the mandate of re-engineering primary healthcare services [8].

The processes that should be involved in the collaboration were outlined in the study, including developing training programs for TBAs. A study conducted in Guatemala confirms that a curriculum should be developed to train TBAs on the following aspects: (i) An overview of maternal-infant care; (ii) an introduction to ovulation, fertilisation, and the natural development of pregnancy; (iii) pregnancy complications and the danger signs during pregnancy; (iv) an introduction to the stages of labour and complications; and (v) lastly, neonatal resuscitation and care of the mother during the postnatal period [37]. Another study confirms that the additional roles of TBAs during a partnership with midwives include breastfeeding health education, postnatal check-ups, advice for women to attend clinics, and conducting health promotion programmes in the community, as supported by [38,39].

In terms of the outcomes, this paper suggests that the inclusion of TBAs at the primary healthcare level can assist with the promotion of culturally appropriate midwifery care and a reduction in maternal mortality rates. A study confirms the current study’s findings by indicating that TBAs have contributed to a range of successful maternal, neonatal, and child health interventions; however, they are unable to manage obstetric complications [38,40]. In addition, it was noted that after two decades, TBA training was ineffective in reducing maternal mortality; the WHO has focused maternal mortality reduction efforts on the importance of having skilled birth attendants (excluding TBAs) for all deliveries [1,38,39,41]. Not long before, the WHO considered a plethora of interventions that consisted of training and re-training TBAs to improve their skills and competencies in managing uncomplicated deliveries and referring the more difficult deliveries to orthodox health facilities [41]. The Ghanaian health ministry has seen the need to address staff shortages of skilled birth attendants (SBAs) through the integration of the TBAs into the CHW (community health worker) concept as an interim measure until there are enough SBAs [33]. In this county, the CHWs are intermediaries between the community and the formal healthcare system. TBAs offer culturally acceptable care and play an important role in supporting the health of women and newborns and linking women, families, and communities to the formal health system [1].

Practical implications of findings

The strengths of the developed inclusive collaborative framework for midwives and traditional birth attendants for maternal and child healthcare in SA may inform policy considerations. The DoH and the Directorate of African Traditional Medicine indicated the need to recognise the contributions of traditional health professionals. The collaborative framework may guide the legislation on including TBAs as part of the maternal and child healthcare services in rural communities to improve universal access to services [1].

Limitations

Although the inclusive framework for collaboration was developed through a consensus process, the final framework has not been validated. However, the participating stakeholders represented the various key role-players required to implement the collaboration and policy considerations. Furthermore, no challenges were presenting larger data sets, which is usually the case for NGTs.

Recommendations

Based on the success of the NGT in identifying and ranking the priority areas in supporting reasons for the development of the inclusive collaborative framework for maternal and child healthcare, we recommend that the DoH consider developing policies to regulate TBA practice for maternal and child healthcare.

## 5. Conclusions

This paper presented key stakeholders’ views on developing an inclusive framework for collaboration between TBAs and midwives in rural SA. The Western biomedical health system dominates the current healthcare system. The system undermines the pluralistic needs of pregnant women to patronise maternal and child healthcare services from both the Western health system and the traditional biosocial system. Thus, this study recommends an inclusive healthcare system that recognises the role of TBAs. However, they are not fully integrated into the healthcare system but integrated only through training, delivery and legislation. We do not argue for the provision of a unilateral, top-down maternal health service but rather for one that collaborates between the biosocial system of SA and the biomedical system supported by government legislation. Ultimately, the proposed framework aims to create a sustainable and culturally sensitive model that optimises the strengths of midwives and TBAs, fostering improved healthcare delivery in rural communities.

## Figures and Tables

**Table 1:** Nominal group technique (NGT) steps.

Steps	Description of Steps	Time Frame
1	Introduction and explanation of the nominal technique process [28]. The researcher explained the 3 components of the SPO Donabedian framework to orientate the participants.	Lasted 15 min
2	Nominal or Silent Generation of ideas The PI posed the following question to start the workshop: What should a framework for collaboration between the midwives and TBAs for maternal health care services in South Africa entail? Participants were asked to note down the ideas that came to mind of the central question posed. Discussions were prohibited in this step, but the facilitator gave attention to those seeking clarity.	Lasted 30 min
3	Round-robin: Clarification of items and sharing of ideas. Each member of the group was given time to share their ideas with the group. These were grouped according to the structure, process, and outcome of the Donabedian framework as they emerged, and ideas were written down on the A3 flip charts.	Lasted 60 min
4	Discussion and presentation of ideas for consensus The facilitator encouraged questions and discussion during this period. This process was also used as an opportunity to probe the presenters for further explanations, as well as for the wider team to discuss and clarify presented ideas.	Lasted 120 min
5	Ranking of ideas Each stakeholder was to vote and rank the ideas presented. The ranking process followed the strategy [29] of ranking ideas by assigning a value to an idea according to priority. For those who attended Microsoft Teams, polls were used for ranking the ideas according to the order of priority between 1–5.	Lasted 15 min

**Table 2:** Key stakeholders’ characteristics and involvement in maternal and child healthcare.

Institution and Country	Occupation	Role
Tshwane Clinics, South Africa	Registered midwife	Pre-conception, pregnancy, labour, and postpartum care
Private clinic, South Africa	Registered midwife	Pre-conception, pregnancy, labour, and postpartum care
University, South Africa	Midwifery educators Student midwife	Conducting training and updating curriculum for midwifery care
University, Swaziland	Midwifery educators	Conducting midwifery training
Society of Midwives in South Africa	Registered midwife	Engagement activities for health professionals
Traditional Health Organisation (THO)	Coordinator	Regulatory body and council of traditional health practitioners, including TBAs
Soshanguve, South Africa	Traditional birth attendants	Pregnancy, labour, and postdelivery in rural communities
Nigeria	Researchers on traditional health practice	Studies on collaboration between midwives and TBAs
Ghana	Researchers on traditional health practice	Professor with expertise in implementation collaboration between midwives and TBAs
National Department of Health (DoH)	Director of Traditional Medicine, Director of Maternal and Child Healthcare	Policymakers
Rural Community	Civil society	Community representatives
United Nations International Children’s Emergency Fund (UNICEF)	Medical doctor	Health education strategies and advocacy for collaboration
DENOSA	Labour organisations	Justice and legal representations for health professionals

**Table 3:** Ranking results in descending order.

Processes (Items)	Voting and Ranking *n* (%)
Conduct training and capacity building for TBAs on low-risk antenatal, labour, and postpartum care	20 (100%)
Develop standardised guidelines with clear TBA roles for maternity care	15 (75%)
Regulation of practice through policy development	13 (65%)
Quality control measures through the Department of Health, standardised care, and accreditation	13 (65%)
Formulate guidelines for referral pathways for midwives and traditional birth attendants	10 (50%)
Scope of practice of traditional birth attendants	8 (40%)
Establish a database system to record all the registered traditional birth attendants in South Africa	8 (40%)
Conduct community workshops on baby care, high-risk maternal conditions such as pre-eclampsia, HIV, and postpartum haemorrhage, and the road to health booklets	8 (40%)
Meetings, i.e., (engagement platforms with stakeholders and MNM meetings (maternal and neonatal mortality meetings)	3 (15%)
Identify appropriate ways to remunerate traditional birth attendants in line with national health insurance (NHI) policies	3 (15%)
Develop a herbal medicine clarification system recording all herbal medicine used during pregnancy	3 (15%)
Record background information on the origin of traditional practice	0
Criteria to identify pregnant patients at risk, such as pre-eclampsia, and refer patients	0

**Table 4:** Outcomes of the framework.

Outcomes (Items)	Voting and Ranking *n* (%)
Patient-centred care and culturally appropriate care	20 (100%)
Reduction in maternal and neonatal mortality rates	16 (80%)
Strategic alignment with policy framework (NHI and re-engineering of primary healthcare policies	16 (80%)
Improve the acceptance and recognition of traditional health as a legitimate system of healthcare	12 (60%)
Improve access to maternal healthcare services at the community level	11 (55%)
Strengthen knowledge-sharing through joint capacity-building programmes	11 (55%)
Extend the collaboration to SBAs, community representatives, and traditional health practitioners (THPs)	7 (35%)
Foster continuous professional development	5 (25%)
A culture shift in the delivery of healthcare	2 (10%)

## Data Availability

The data presented in this paper are available from the corresponding author upon request.
